# Role of L-carnitine in Cardiovascular Health: Literature Review

**DOI:** 10.7759/cureus.70279

**Published:** 2024-09-26

**Authors:** Ramy Elantary, Samar Othman

**Affiliations:** 1 Department of Acute Medicine, Royal Liverpool University Hospital, Liverpool, GBR; 2 Department of Diabetes and Endocrinology, Countess of Chester Hospital, Chester, GBR

**Keywords:** diet and cardiovascular health, role of l-carnitine, primary prevention, cardiovascular health, l-carnitine

## Abstract

Cardiovascular diseases (CVDs) remain the leading cause of morbidity and mortality worldwide. Secondary preventive measures, like anti-platelet medications, B-blockers, and angiotensin-converting enzyme (ACE) inhibitors, have been found to dramatically lower the risk of cardiovascular disease. However, prolonged usage of these drugs has been linked to multiple adverse impacts. Hence, finding more efficient treatments, especially dietary strategies for long-term use in daily life, is advantageous for primary prevention and treatment. L-carnitine, a naturally occurring amino acid derivative normally synthesized in the liver and kidney, is believed to have a considerable influence on cardiovascular health. L-carnitine can enhance both contractile performance and structural integrity of the cardiac muscle by maintaining efficient energy production and reducing oxidative stress.

This literature review aims to address several pressing questions regarding the role of L-carnitine in cardiovascular health: what are the physiological functions of L-carnitine, particularly in relation to cardiovascular health; how effective and safe is L-carnitine supplementation in the management of various cardiovascular diseases, primarily ischemic heart disease, heart failure, and peripheral vascular disease; what are the underlying mechanisms through which L-carnitine exerts its cardioprotective effects; what controversies exist in the current research; and finally, what should be the future directions? Through this comprehensive analysis, the review aims to enrich our understanding of L-carnitine's role in cardiovascular health, providing a robust foundation for future academic and clinical endeavors.

PubMed, Embase, and Google Scholar have been used to search the following keywords: L-carnitine, cardiovascular health, mitochondrial function, and L-carnitine side effects. Then, using the existing search engine formats, some keyword combinations were used to find the related articles included and every possibility, including using every first keyword combination with another keyword, using every keyword in every place at each given box, etc. Around 308 articles were reviewed using this process, including systemic reviews, meta-analysis studies, randomized controlled trials, and literature review articles. In the end, after leaving the pure articles related to the topic as 35 articles, which are attached below with direct citation, the majority of them were very fresh articles, as recent as 2010, and back words, except just one paper related to the impact of L-carnitine post-myocardial infarction, as its data provided us with a positive and promising impact of L-carnitine in this field.

L‑carnitine seems to have a pivotal role in cardiovascular health due to its energy metabolism, anti-oxidative stress, and endothelial role. The safety and effectiveness of L-carnitine administration remain an issue for scientific investigation. One of the major concerns is that the intestinal metabolism of L-carnitine generates trimethylamine-N-oxide (TMAO), a compound that has been linked with faster atherosclerosis progression.

## Introduction and background

Cardiovascular diseases have become the leading cause of mortality and morbidity the world over [1,2,3,4]. The alarming prevalence of cardiovascular diseases necessitates the development of potent therapies and interventions.

No one can deny that secondary prevention agents for cardiovascular disorders, such as anti-platelets and angiotensin-converting enzyme inhibitors, have proven beneficial in reducing both morbidity and mortality. On the other hand, long-term use of these is also associated with a lot of side effects in the form of electrolyte derangement and bleeding. Thus, the discovery of a natural compound like L-carnitine, which may have similar beneficial effects as secondary or primary care with fewer adverse events, shall be beneficial.

L-carnitine, a derivative of amino acids, has attracted immense interest for its effect on cardiovascular diseases. L-carnitine is a naturally occurring amino acid that is synthesized mainly in the liver and kidneys and is needed for transporting long-chain fatty acids into mitochondria, where they are further broken down for beta-oxidation to release energy [5]. Along with its significant function in fat metabolism, it has been proven that L-carnitine can operate as an antioxidant (scavenger of free radicals) and anti-inflammatory agent, shielding tissues from harm produced by reactive oxygen species [6]. The lipid-lowering effect, anti-inflammatory properties, and ability to reduce ischemic-related injury are among the mechanisms through which L-carnitine exerts its beneficial effects in various cardiovascular diseases.

L-carnitine can be generally considered a safe supplement. However, its therapeutic effect on cardiovascular disease remains a topic of considerable debate and controversy [7]. On one hand, many studies have supported a cardioprotective role for L-carnitine [8,9,10]. On the other hand, given the worrisome concern regarding the potential atherosclerotic effect of its metabolite trimethylamine N-oxide (TMAO) [7,11], some have questioned the safety of L-carnitine.

Despite a series of positive findings on the beneficial therapeutic target for various cardiovascular diseases derived from the evidence from clinical trials, there is still an urgent need for more meaningful, well-designed, randomized controlled trials to better define cardiovascular pharmacotherapy, the safe window of L-carnitine, and the longer-term side effects, including potential atherosclerotic effects.

This literature review concentrates on the magnitude of cardiovascular diseases in the global population, the mechanism of L-carnitine in cardiovascular function and its possible effects in different cardiovascular diseases, the side effects and risks of its supplementation, debates, and controversies on its role, and future directions in the context of currently gathered research and applications.

## Review

Mechanisms of action of L-carnitine in cardiovascular health

L-carnitine helps with energy production by breaking down fat, enhancing the efficiency of mitochondria, reducing oxidative stress and inflammation, and altering lipid concentrations due to the reduction of fats in the bloodstream. L-carnitine is, therefore, bestowed to oversee the proper functioning of the heart and blood vessel systems. The above-mentioned mechanisms are better observed in field cases of variable heart conditions such as ischemic heart disease and heart failure. This will be further expatiated in the subsequent sections.

Role of L-carnitine in Mitochondrial Function and Energy Production

L-carnitine supports mitochondrial function and energy production in multiple ways. The most crucial mechanism is shuttling fatty acids through the mitochondrial membrane to be used for energy production. L-carnitine is the only known molecule that will enable fatty acids to cross the inner membrane and enter the mitochondria, where they are oxidatively degraded, a process called B-oxidation [12]. A system of enzymes and transporters allows the L-carnitine molecule to cross the cell membrane and then carry the fatty acid molecules across the inner mitochondrial membrane.

**Figure 1:**
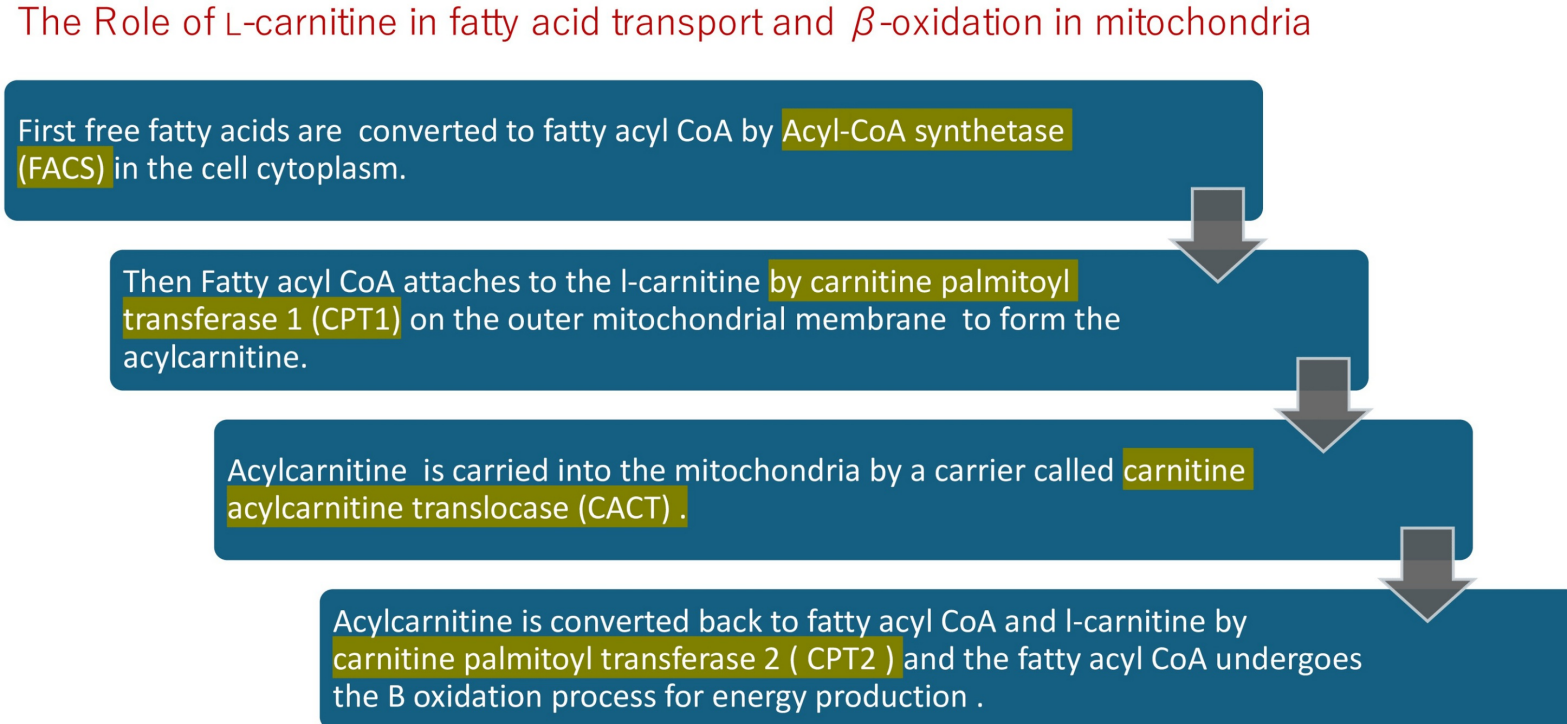
The role of L-carnitine in fatty acid transport and β-oxidation in mitochondria CoA: coenzyme A Image credits: Elantary Ramy, Othman Samar

The fatty acid molecules must first be converted to fatty acyl CoA by binding with coenzyme A (CoA) in the first step of the beta-oxidation process. These fatty acyl CoA molecules are then shuttled into the mitochondria, where they bind to L-carnitines to form acylcarnitines. Acylcarnitines convert back to fatty acyl CoA within the mitochondria matrix. The fatty acyl CoA molecules are again capable of the beta β-oxidation sequence. Beta-oxidation produces acetyl fragments from the fatty acid molecule in the form of acetyl-coenzyme A (acetyl CoA), which enters the Krebs (citric acid) cycle and participates in the formation of adenosine triphosphate (ATP) energy (Figure 1) [13].

Moreover, there is strong evidence that L-carnitine impacts mitophagy and mitochondrial biogenesis, which are both vital to mitochondrial quality control. Mitophagy describes the selective degradation of impaired or redundant mitochondria, whereas mitochondrial biogenesis refers to the production of novel mitochondria. Evidence has shown that L-carnitine can promote mitochondrial biogenesis by upregulating peroxisome proliferator-activated receptor-gamma coactivator-1alpha (PGC-1α), a master regulator of mitochondrial biogenesis [14] and that L-carnitine promotes mitochondrial protein acetylation, a chemical modification that improves mitochondrial function and protects against stressors. By promoting such processes, it has now become evident that L-carnitine can preserve a heterogeneous population of healthy mitochondria to maintain a remarkable production of cellular energy.

There is another pathway by which L-carnitine assists the mitochondria to remain healthy. This is because it helps to preserve the membrane potential of mitochondria. One of the most critical features of the mitochondria to maintain ATP synthesis lies in its ability to preserve the membrane potential, a difference in electrical charge between the mitochondrial matrix and the rest of the cell cytoplasm. L-carnitine places the negative electrical charge of phospholipids on the inside of the mitochondrial membrane and the positive electrical charge of the protein on the outside portion of the mitochondrial membrane. L-carnitine does this by interacting with both the membrane phospholipids and their proteins, thus preventing the loss of mitochondrial membrane integrity. Preserving the membrane potential of the mitochondria through carnitine is vital to cellular health because if the membrane potential collapses, then apoptosis or other forms of cell death will occur [15,16].

Finally, L-carnitine is involved in the composition and regulation of acetyl-CoA, one of the most important intermediaries of the metabolism. The balance between acetyl-CoA and coenzyme A (or CoA) is determined by L-carnitine, which is instrumental in the conversion of acetyl-CoA to acetyl-L-carnitine, which can be then dissipated out of the mitochondria, thus avoiding the accumulation of acyl groups inside the mitochondria, which could be toxic. In this way, we maintain the balance of metabolic flexibility and guarantee enough free CoA available to sustain other metabolic reactions [17].

Antioxidant Properties

Research has demonstrated that higher levels of oxidative stress play an important role in the development of coronary artery disease (CAD) [8].

Reactive oxygen species (ROS) are high-reactivity oxygen species derived from the reduction of oxygen [18]. The family includes both free radical species (e.g., superoxide (O2·−) or hydroxyl species (HO·)) as well as non-free radical species (e.g., hydrogen peroxide (H2O2)) or more complex species that can derive from them, such as peroxynitrite (ONOO-) and hypochlorous acid (HOCl). Physiological values of ROS are required to sustain the signaling pathways controlling crucial cellular processes, among which are inflammation, differentiation, proliferation, homeostasis, and apoptosis, whereas the generation of dysregulated ROS can amplify these same pathways, thus fostering disease phenotypes associated with ROS, as seen in type II diabetes, cancer, and atherosclerosis [19]. Furthermore, elevation of ROS production is per se an important risk for additional ROS generation due to the presence of ROS-induced ROS release systems that can speed further illnesses [19].

L-carnitine plays a particularly important role in several critical pathways that amplify its antioxidant activity. A major route of action is controlling fatty acid metabolism, as previously mentioned. By helping to convey long-chain fatty acids into the mitochondria for energy production, it prevents their accumulation. By keeping fatty acids from accumulating, oxidative stress is avoided, protecting the cellular equilibrium and helping to prevent damage from oxidation.

Further mechanism exerted by L-carnitine is based on the quenching of reactive oxygen species (ROS) and reactive nitrogen species (RNS) [9]. It upregulates the activity and the expression of antioxidant enzymes such as superoxide dismutase (SOD), catalase, and glutathione peroxidase, increasing the conversion of superoxide radicals into less reactive species and decreasing the oxidative stress [18].

Another mechanism [20] is the stabilization of cell membranes since oxidative stress induces lipid peroxidation, which destabilizes the cell membranes, provoking damage and apoptosis. L-carnitine reduces lipid peroxidation through the scavenging of free radicals and the enhancement of the mitochondrial membrane structural integrity.

Furthermore, L-carnitine alters the expression of genes mediated by oxidative stress, including the Nrf2 pathway. Nrf2 is activated by oxidative stress and increases the expression of numerous antioxidant response elements (AREs) and therefore the antioxidant capacity of the cell [21].

**Figure 2:**
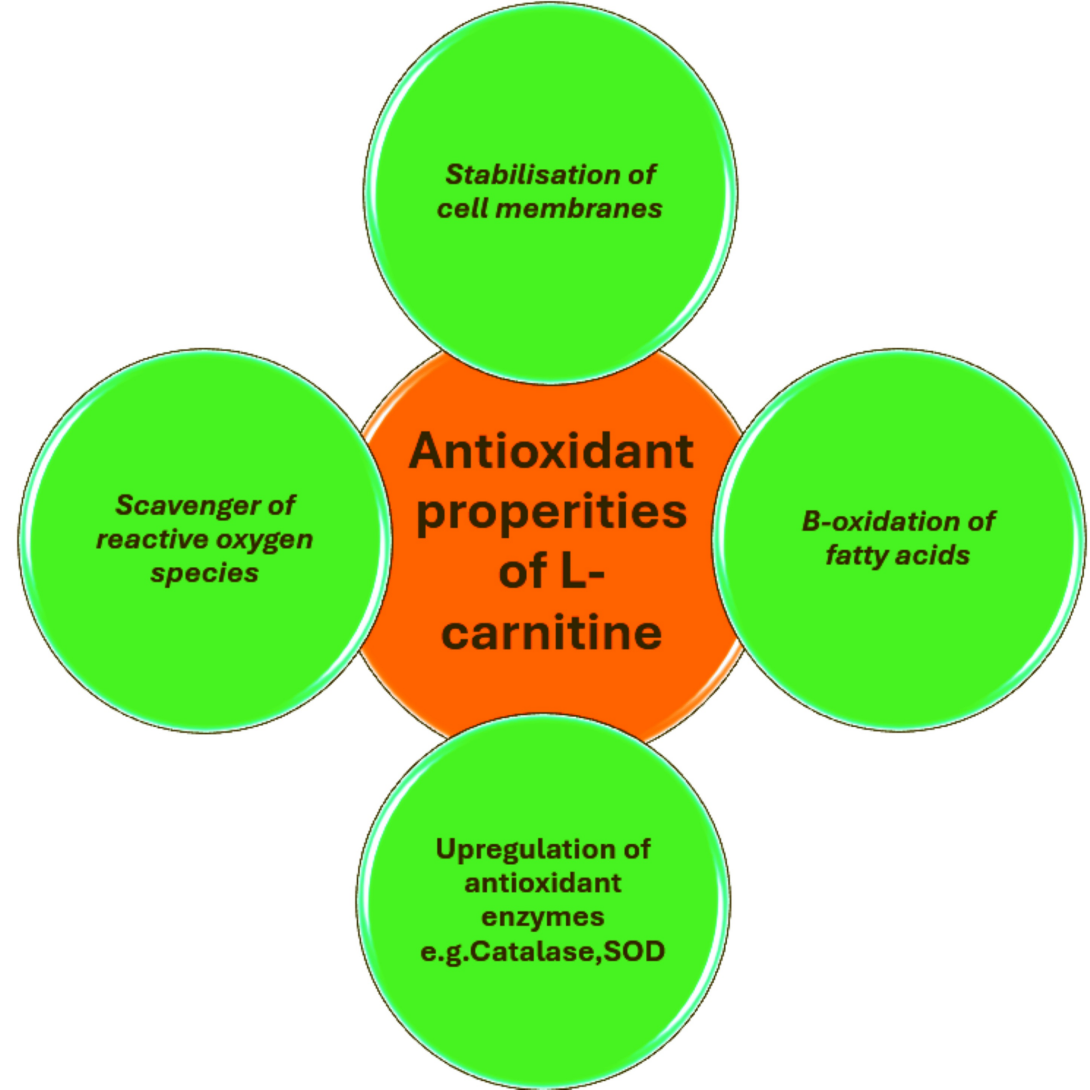
Anti-oxidant properties of L-carnitine SOD: superoxide dismutase Image credits: Elantary Ramy

Finally, L-carnitine is involved in boosting the cellular antioxidant defense system by enhancing the action of the cell’s naturally produced antioxidants (such as glutathione and vitamin E) through regeneration and recycling [22,23]. This synergistic reinforcement increases the cell’s antioxidant reserves and protects the cell from oxidative damage and loss of integrity (Figure 2).

Anti-inflammatory Properties

Many theories describing the atherosclerotic disease process are correlated with inflammation status. Mounting evidence indicates that a higher inflammation status plays a key role in the development of coronary artery disease (CAD) [24, 25]. L-carnitine exhibits anti-inflammatory activity by modulating several biochemical pathways and pharmacodynamic interactions with pro-inflammatory cytokines.

Firstly, L-carnitine exerts potent anti-inflammatory effects via its action as an antioxidant and as a main compound in mitochondrial action and health, as previously discussed.

Another important mechanism [22] involves its ability to inhibit nuclear factor kappa B (NF-κB), a transcription factor responsible for the transcription and production of multiple pro-inflammatory cytokines, such as tumor necrosis factor-alpha (TNF-α), IL-1β, and IL-6. Reduced NF-κB-induced activation of these genes reduces inflammation.

Further molecular mechanism by which L-carnitine reduces inflammation involves the induction of peroxisome proliferator-activated receptor-γ (PPAR-γ), a receptor that suppresses the expression of inflammatory genes and promotes the resolution of inflammation [9].

**Figure 3:**
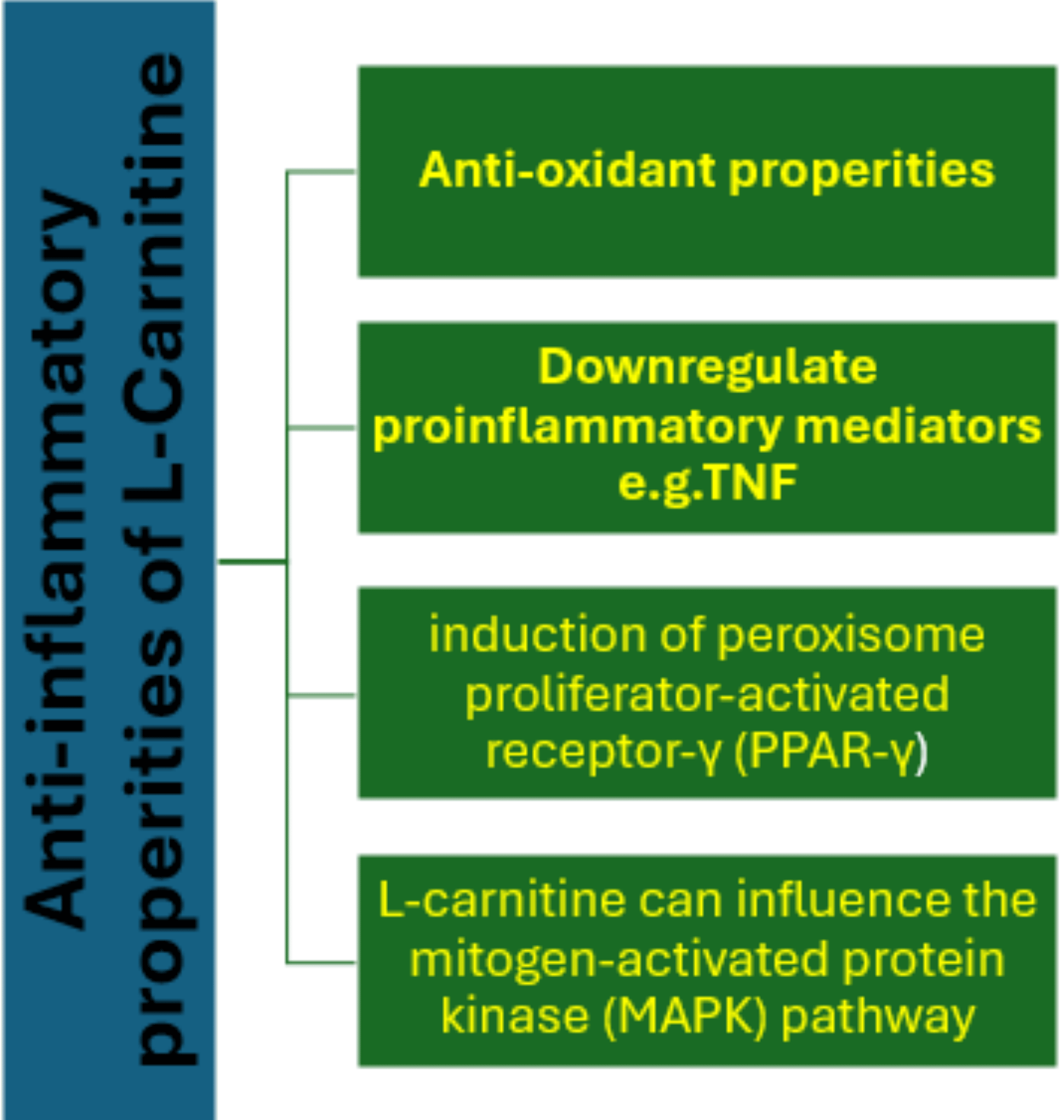
Anti-inflammatory properties of L-carnitine TNF:  tumor necrosis factor Image credits: Othman Samar

Finally, L-carnitine can influence the mitogen-activated protein kinase (MAPK) pathway, which is involved in the regulation of cell proliferation, differentiation, and inflammatory responses. The inhibitory effects of L-carnitine on MAPK phosphorylation provide for a decreased production of pro-inflammatory cytokines (Figure 3) [26].

With the physiological effects of L-carnitine listed above, its importance for cardiovascular health is evident. This key compound supports energy metabolism by facilitating fatty acid oxidation and optimizing mitochondrial function, minimizing oxidative stress and inflammation, and altering lipid profiles by reducing circulating lipids. Hence, L-carnitine substantially contributes to the proper function of the heart and vascular system. The beneficial effects of L-carnitine demonstrate the utmost importance of this amino acid in the maintenance of cardiovascular health and underpin its potential therapeutic use in the management of cardiovascular disease and disease prevention.

Clinical effects of L-carnitine in different cardiovascular diseases

Effect of L-carnitine on Heart Failure

Heart failure is a condition that, despite enormous modern advances in medicine and technology, is still a devastating disability that burdens millions of people across the globe. It occurs when the heart cannot pump enough blood to meet the body’s needs and is a cardiac disease that results in major morbidity and mortality. The available treatments are all symptomatic, slowing disease progression and improving patient quality of life. The standard treatments are angiotensin-converting enzyme (ACE) inhibitors, angiotensin receptor neprilysin inhibitors (ARNIs), sodium-glucose co-transporter-2 inhibitors (SGLT2 inhibitors), beta-blockers, diuretics, and aldosterone antagonists [27]. All address different pathologic elements of the disease, including lowering preload and afterload, limiting activation of neurohormonal pathways, and improving cardiac contractility. Although these methods have proven clinical efficacy, they fail to directly target the central pathophysiologic mechanisms at play in heart failure.

Literature suggests that L-carnitine supplementation increases heart energy output, improves left ventricular function, and reduces myocardial injury in different animal models of heart failure. L-carnitine was also effective in reducing cardiac remodeling, and oxidative stress, and altering neurohormonal pathways known to worsen heart failure. A meta-analysis of randomized controlled trials, performed by Xiaolong Song et al. in 2017, examined the effectiveness and tolerance of L-carnitine (L-C) supplements in patients with chronic heart failure (CHF) [10]. In total, 17 randomized controlled trials consisting of 1625 heart failure patients receiving L-C were included in this meta-analysis. The present data suggested that L-C treatment for CHF showed significant improvement in the overall efficacy (OR = 3.47, P <0.01), left ventricular ejection fraction (LVEF) (WMD: 4.14%, P = 0.01), stroke volume (SV) (WMD: 8.21 ml, P = 0.01), cardiac output (CO) (WMD: 0.88 L/min, P <0.01), and E/A (WMD: 0.23, P <0.01). Meanwhile, L-C treatment resulted in a significant decrease in the serum levels of brain natriuretic peptide (BNP) (WMD: −124.60 pg./ml, P = 0.01), serum levels of NT-proBNP (WMD: −510.36 pg./ml, P <0.01), left ventricular end-systolic diameter (LVESD) (WMD: −4.06 mm, P <0.01), left ventricular end-diastolic diameter (LVEDD) (WMD: −4.79 mm, P <0.01), and left ventricular end-systolic volume (LVESV) (WMD: −20.16 ml, 95% CI: −35.65 to −4.67, P <0.01). However, there were no significant differences in all-cause mortality (OR = 0.48, 95% CI: 0.21 to 1.06, P = 0.07), six-minute walk (WMD: 45.41 m, 95% CI: −14.46 to 105.29, P = 0.14), and adverse events between L-C and control groups (5.4% versus 5.8%, OR = 0.92, 95% CI: 0.44 to 1.92, P = 0.83). Three limitations of this meta-analysis need to be pointed out. The first one is the less optimal methodological quality of the included studies, so bias cannot be excluded. The second one is the low number of patients in the included trials (only 1625 patients from 17 RCTs). Thus, more RCTs should be carried out in the future to evaluate the effect of L-C on CHF patients. The third one is that the follow-up duration for these studies ranged from seven days to three years, which may result in potential publication bias and reduce the reliability of these trials. In conclusion, this meta-analysis confirmed that L-carnitine supplements can increase LVEF, stroke volume, cardiac output, decrease left ventricular end-diastolic diameter (LVEDD), left ventricular end-systolic diameter (LVESD), and left ventricular end-systolic volume (LVESV), serum levels of BNP and NT-proBNP, as well as a satisfactory safety.

Some reports [28,29] suggested that clinical symptoms, cardiac function, and renal function in CHF patients with renal insufficiency may be more likely to be ameliorated with L-carnitine treatment.

In line with the ‘energy starvation’ hypothesis [30], which induced that energy supply scarcity was responsible for the contractile dysfunction in heart failure. An integral part of the ‘energy starvation’ hypothesis is mitochondrial dysfunction. The mitochondria is the predominant production site for ATP in cardiac cells. An obvious abnormality in heart failure is a reduction in mitochondrial density and function leading to reduced ATP production. This ATP deficiency disturbs multiple ATP-dependent processes, such as electrolyte transport, substrate utilization, and contractile protein interactions, resulting in impaired myocardial contractility. Another contributing factor is impaired substrate metabolism. The heart predominantly uses fatty acid fuels for energy production. In heart failure, there is a metabolic switch towards higher fat oxidation and glucose utilization. The partitioning of substrate utilization is less efficient and adds to the injury caused by an energy deficit. Furthermore, oxidative stress and hence free radical damage to mitochondria and other cellular structures deplete cardiomyocyte energy stores. Levels of reactive oxygen species (ROS) produced during the stress impair mitochondrial DNA and result in dysfunctional electron transport chains and reduced ATP synthesis. Based on this hypothesis, it can now be understood how L-carnitine exerts its effects in heart failure patients.

Further beneficial effects exhibited by L-C administration have been described, such as oxidative stress [31], nitric oxide [32], arterial hypertension, cardiac inflammation and fibrosis [9], and interstitial remodeling, as well as the improvement of endothelial function [33].

Therefore, the combined results from several heart failure clinical trials and studies indicate that L-carnitine could be an effective agent for the treatment of heart failure, improving cardiac function, physical activity, and the patient's quality of life. Although the evidence is promising, more research is needed to establish all the beneficial effects of L-carnitine, as previously mentioned. 

Role of L-Carnitine in Atherosclerosis and Coronary Artery Disease

Coronary artery disease (CAD) is still one of the most common causes of death and extends to a significant amount of morbidity and mortality. Over the recent decades, a lot of advancements have been and are being made in the field of medicine and science to develop efficient treatments for coronary artery disease.

Management of CAD includes lifestyle modifications, pharmacotherapy to reduce the risk factors of CAD, and interventional procedures. Lifestyle modifications include changes in diet, an increase in physical activity, and smoking cessation. Pharmacotherapy often includes anti-platelet agents such as aspirin, statins to control cholesterol, beta-blockers, and angiotensin-converting enzyme inhibitors to control blood pressure. In the advanced cases of CAD, interventional procedures such as percutaneous coronary intervention (PCI) or coronary artery bypass grafting (CABG) are employed. In PCI, the narrowed coronary artery is opened by placing stents, whereas in CABG, a bypass is created around the blocked artery using a vessel from another part of the body.

However, despite the many advances in CAD treatment, the disease is invariably associated with many limitations, most significantly the comorbid conditions that complicate treatment protocols and patients’ quality of life; the significant lifestyle modifications that patients must follow to obtain desired outcomes, which are difficult to sustain over long periods; the side-effect burdens of pharmacotherapy and possible drug interactions; the risks of interventional procedures, especially restenosis (or re-narrowing) of treated arteries and adverse events occurring during the preoperative and postsurgical periods; the economic burden created by these advanced treatments, which compromises some patients access to quality care; and the individual variability in treatment responses, which necessitates continued research and development.

L-carnitine is considered one of the most promising pharmacological agents in the treatment of CAD. Multiple studies have shown that higher oxidative stress leads to CAD, so administering positive antioxidants to CAD patients will improve prognosis and prevent the recurrence of CAD [34], hence the potential effect of L-carnitine in these patients.

A randomized, placebo-controlled trial by Bor-Jen Lee et al. studied the effects of L-carnitine supplementation on oxidative stress and antioxidant enzyme activities in patients with coronary artery disease [35]. In this trial, the oxidative stress in patients with CAD has been reduced significantly by L-carnitine administered at a dose of 1000 mg/d for 12 weeks with the increase in the activities of antioxidant enzymes. Forty-seven CAD patients were enrolled in the study. CAD patients were considered to have the presence of at least 50% stenosis of one major coronary artery through cardiac catheterization of the coronary arteries. The patients were randomly assigned with 24 in the placebo group (PL) and 23 in the L-carnitine group (L-C), and the intervention period lasted 12 weeks. Levels of serum L-carnitine, plasma malondialdehyde (MDA), and erythrocyte antioxidant enzyme activities (catalase (CAT), superoxide dismutase (SOD), glutathione peroxidase (GPx)) were determined before and after intervention.

Thirty-nine subjects completed the study (placebo, n = 19; L-carnitine, n = 20). During the process of 12-week L-carnitine supplementation, the level of MDA was significantly decreased (2.0 ± 0.3 to 1.8 ± 0.3 μmol/L, P = 0.02), and meanwhile, the mean level of L-carnitine (33.6 ± 13.6 to 40.0 ± 12.0 μmol/L, P = 0.04), as well as antioxidant enzyme activities (CAT (12.7 ± 5.5 to 13.1 ± 5.8 U/mg of protein, P = 0.02), SOD (14.8 ± 2.9 to 20.7 ± 5.8 U/mg of protein, P < 0.01), and GPx (20.3 ± 3.4 to 23.0 ± 3.1 U/mg of protein, P = 0.01)), were significantly increased. The level of L-carnitine was significantly positively correlated with the antioxidant enzyme activities (CAT, β = 0.87, P = 0.02; SOD, β = 0.72, P < 0.01). Importantly, some limitations of this study need to be addressed. Firstly, this study was conducted using a relatively small number of participants. Secondly, this study was modeled on daily L-C supplements for three months only. Therefore, to properly improve and establish the clinical beneficial effects of high doses of L-C in CAD patients, further long-term and large-scale intervention studies are needed.

It is obvious now that L-carnitine has a potential cardioprotective effect against CAD, which its antioxidant property may justify [36]. This protective effect can also be explained through its role in fatty acid metabolism and improving the lipid profile of coronary artery disease patients. A further randomized, placebo-controlled trial by Bor-Jen Lee et al. studied the effects of L-carnitine supplementation on lipid profiles in patients with coronary artery disease [37]. A total of 47 subjects were enrolled and randomly assigned to the placebo group (n = 24) and to the L-carnitine group (n = 23). The duration of the intervention was 12 weeks. Changes in L-carnitine levels, lipid profiles, and antioxidant enzyme activity (superoxide dismutase (SOD)) were examined. At week 12, subjects in the L-carnitine group showed higher SOD activity (20.7 ± 4.2 versus 13.1 ± 2.9 U/mg of protein, P < 0.01), high-density lipoprotein cholesterol (HDL-C) (1.34 ± 0.42 vs. 1.16 ± 0.24 mmol/L, HDL-C, P = 0.03), and apolipoprotein A1 (Apo-A1) (1.24 ± 0.18 vs. 1.12 ± 0.13 g/L, P = 0.02) than those in the placebo group. Triglyceride (TG) (1.40 ± 0.74 vs. 1.35 ± 0.62 mmol/L, P = 0.06) showed a slight significance. Besides, L-carnitine level was negatively correlated with TG and Apo-B and positively correlated with HDL-C and Apo-A1 after supplementation. At the same time, SOD activity was highly significantly inversely correlated with lipid profiles (total cholesterol, TG, and Apo-B) after supplementation. Therefore, 1000 mg/d of L-carnitine supplementation brought about a significant increase in HDL-C and Apo-A1 levels and a slight decrease in TG levels in CAD patients. It did not produce any other changes in lipid profile.

One advantage of this study is that it is the first clinical study to clarify the L-carnitine lipid-lowering effect in CAD patients, and it measured L-carnitine levels, lipid profiles, and activity of antioxidant enzymes. This study provides direct evidence to clarify the correlation among the L-carnitine level, lipid profile, and antioxidant ability; however, further long-term intervention studies with a larger sample size may be needed to clarify lipid-lowering effects after L-carnitine intervention in patients with hyperlipidemia. Some limitations of the present study should be noted. First, the number of subjects was small; however, it did calculations to examine statistical power for lipid profiles. The statistical power of the differences of TG, HDL-C, and Apo-A1 was 0.97, 0.86, and 0.86, respectively. Second, some subjects in the L-carnitine group had hyperlipidemia; thus, it may not be able to detect the lipid-lowering effects in the above subjects. Third, experimental results indicate that there may be potential lipid-lowering effects of L-carnitine intervention; however, an optimal dose of L-carnitine for lipid-lowering effect should be defined.

It is worth mentioning that there is some research work suggesting that dietary L-carnitine might accelerate atherosclerosis via gut microbiota metabolites. Choline and L-carnitine are broken down to the disease-linked metabolite, trimethylamine (TMA), by the gut microbiota. The TMA is transported in the portal circulation to the liver, where flavin monooxygenases (FMOs) synthesize trimethylamine-N-oxidase (TMAO) from TMA [38]. This small molecule, known as TMAO, has received attention in recent years, with various lines of evidence suggesting a correlation between a higher risk of CVDs and all-cause mortality [39, 40]. Obviously, this makes the role of L-carnitine supplements in health a little more complex [41]. You can read more about this in the part about side effects of L-carnitine.

In conclusion, L-carnitine supplementation can effectively attenuate oxidative stress and lipid profile in CAD patients and can promote the activities of antioxidant enzymes. L-carnitine supplements may be useful in improving the antioxidant capability of CAD patients, therefore improving the prognosis and outcome in such patients. Despite that, further larger studies need to be conducted to address the debates around its use, especially the potential atherosclerotic effect of its metabolite, TMAO.

Use of L-carnitine in Post-Myocardial Infarction Therapy/Secondary Prevention of Cardiovascular Disease

The heart muscle consists of cardiomyocytes, capillaries, and extracellular matrix (ECM), which consists of different types of collagen fibers with important structural attributes, providing strength to the heart muscle. The mechanics of the left ventricle (LV) dictate the hemodynamics of the whole organism. ‘Remodeling’ of the left ventricle (LV) refers to the adaptation of the heart pathologically to mechanical, neurohormonal, as well as genetic changes, the regulation of its dimension, surface structure, and function. Whereas cardiomyocyte hyperplasia brought about by increased microcirculatory blood supply (e.g., during pregnancy, growth, or when undergoing athletic training) is considered physiological and perfectly reversible, ‘adverse’ or ‘pathological’ remodeling following myocardial infarction (MI) is associated with disproportionate risk for heart failure (HF) and carries significant worse survival [42].

Therefore, prevention of left ventricular remodeling is a major therapeutic goal after acute myocardial infarction. A lot of randomized, placebo-controlled trials have qualitatively demonstrated that angiotensin-converting enzyme inhibitor (ACEI)/angiotensin receptor blocker (ARB) therapy prevents or at least limits the remodeling of the left ventricle after myocardial infarction [43]. However, although angiotensin-converting enzyme inhibitors are effective, they need to be stopped in many patients due to adverse events, including symptomatic hypotension, kidney failure, cough, diarrhea, and dizziness.

Despite being a relatively old study, the L-carnitine Ecocardiografia Digitalizzata Infarct Miocardico (CEDIM) trial [44] showed how effective L-carnitine is post-myocardial infarction. The CEDIM trial was a randomized, double-blind, placebo-controlled, multicenter trial in which 472 patients with acute myocardial infarction and high-quality two-dimensional echocardiograms received either placebo (239 patients) or L-carnitine (233 patients) within 24 hours of chest pain onset. L-carnitine was given intravenously at a dose of 9 g/day for the first five days and then 6 g/day orally for the next 12 months. Echocardiograms were evaluated on admission, at discharge from the hospital, and at three, six, and 12 months after acute myocardial infarction. The results from the CEDIM trial showed that early and prolonged L-carnitine therapy in acute myocardial infarction attenuates progressive left ventricular dilation. People treated with L-carnitine had a significantly less pronounced percent increase in both end-diastolic volume (EDDV) and end-systolic volume (ESDV) at three months (EDDV: 18.0 +/- 2.5% vs. 11.1 +/- 2.2%; end-systolic volume 22.5 +/- 3.2% vs. 12.6 +/- 3.1% (placebo vs. L-carnitine)); six months (end-diastolic volume 19.5 +/- 2.3% vs. 12.7 +/-2.1%; end-systolic volume 25.5 +/- 3.2% vs. 15.1 +/-9.8% (placebo and L-carnitine); and 12 months (end-diastolic volume 28.5 +/‐ 3.1 percent vs 19.1 +/- 2.7 percent; end-systolic volume 39.9 +- 4.2 percent vs 28.9 + _ 3.9 percent (placebo vs L-carnitine)) after the emergent event.

Further systematic review and meta-analysis of 13 controlled trials (N=3629) was conducted by James J. DiNicolantonio et al. to determine the effects of L-carnitine vs. placebo or control on mortality, ventricular arrhythmias (VAs), angina, heart failure, and reinfarction [6]. This systemic review suggests that L-carnitine reduces all-cause mortality and ventricular arrhythmia as well as the frequency of anginal attacks. Overall, L-carnitine was associated with impressive 27 percent reduction in all-cause mortality (odds ratio 0.73; 95 percent CI 0.54-0.99; P=0.05; risk ratio 0.78; 95 percent CI 0.60-1.00; P=0.05), highly significant 65 percent reduction in VAs (risk ratio 0.35; 95 percent CI 0.21-0.58; P<0.0001), and significant 40 percent reduction in the development of angina (risk ratio 0.60; 95 percent CI 0.50-0.72; P<0.00001), with no reduction in the development of heart failure (risk ratio 0.85; 95 percent CI 0.67-1.09; P=0.21) and myocardial reinfarction (risk ratio 0.78; 95 percent CI 0.41-1.48; P=0.45).

Current guidelines for acute coronary syndrome (ACS)/acute myocardial infarction (AMI) do not include L-carnitine, but as just reviewed in this systemic review, the evidence is far from weak and necessitates a larger multicenter trial to determine if indeed this simple, cost-effective, and safe agent will benefit patients suffering from acute myocardial function and perhaps also those with stable angina.

The possible mechanisms that account for the beneficial response seen with L-carnitine in AMI are probably multifactorial and include, in part, an improvement in myocardial mitochondrial energy metabolism in the heart due to its ability to enhance the transport of long-chain fatty acids from the cytosol to the mitochondrial matrix where β-oxidation occurs, removing toxic fatty acid intermediates, lessening ischemia secondary to elevated long-chain fatty acid concentrations, and replenishing the depleted carnitine concentrations seen in ischemic, infarct, and failing myocardium. L-carnitine also exhibits beneficial effects on LV remodeling, leading to a significant reduction in LV volumes after AMI, as previously discussed in the CEDIM trial. A clear reduction in infarct size (measured by reductions in cardiac enzymes) has been observed in several AMI clinical trials [45,46], leading to improvements in myocardial viability and salvage. L-carnitine has also been shown to significantly reduce ventricular arrhythmias after acute myocardial infarction, which may well be one of the mechanisms by which it provides an early life-saving beneficial response, explaining the early significant 39% reduction in five-day mortality that was prespecified as a secondary endpoint in the Carnitine Ecocardiografia Digitalizzata Infarct Miocardico 2 (CEDIM 2) [47].

In conclusion, the potential benefit of L-carnitine treatment of patients with acute myocardial infarction can represent the conceptual basis for a larger-scale trial specifically designed and aimed at evaluating the long-term clinical impact of L-carnitine and its side effects.

Impact of L-Carnitine on Peripheral Vascular Disease

Peripheral vascular disease (PVD) is marked by progressive narrowing of arteries throughout the body. It increases the risk of amputation and death and can severely curtail one’s quality of life. A widely accepted international classification of peripheral artery disease (PAD) is Fontaine’s classification (Fontaine 1954); asymptomatic patients are stage I, those with intermittent claudication (IC) are stage II, with rest pain stage III, and with trophic lesions stage IV [48].

Intermittent claudication is the most characteristic symptom of lower extremity PAD. It results from atherosclerosis in the peripheral arteries of the legs, which causes an insufficient arterial blood supply during exercise, resulting in anaerobic metabolism in the muscles with the production of lactic acid and other metabolites. When PAD progresses to the advanced disease stage, the ischemic injury becomes more severe, manifesting as rest pain, chronic ulceration, gangrene, and finally loss of tissue. Intermittent claudication is a warning of systemic atherosclerosis and is considered an independent risk factor for cardiovascular morbidity and mortality [48].

Management strategies for symptomatic and asymptomatic PAD include maximal risk factor modification and conservative treatment with exercise. Symptomatic treatment for PAD adds maximal risk factor modification, exercise, and, when indicated, invasive treatment using balloon angioplasty with or without stenting and bypass surgery together. Today, drugs are known with proven efficacy for the prevention of major cardiovascular and cerebrovascular events: antiplatelets, ACE inhibitors, and lipid-lowering drugs (mainly statins, which additionally have a proven benefit for walking distance) [48]. The only FDA-approved medical therapies for IC are pentoxifylline, cilostazol, and a phosphodiesterase III (PDE3) inhibitor [49].

The main biochemical mechanism through which L-carnitine acts in PVD is related to its established role in fatty acid metabolism. This means that the rate of fatty acid oxidation could be maintained while glucose utilization decreased, and muscle glycogen could be maintained for maximal rates of oxidative ATP production and avoidance of muscular fatigue [50].

Moreover, L-carnitine may also modulate endothelial function and nitric oxide (NO) production. Endothelial cells lining the blood vessels are responsible for releasing NO, a major vasodilator, whose highest levels are produced during high blood flow to optimize vascular tone and reduce vascular resistance. By what is believed to be normal intracellular mechanisms, NO enhances the tolerance to low nutrient levels by dilating the blood vessels, improving blood flow, and thus maximizing the oxygen supply to tissues. It has been demonstrated that L-carnitine supplementation can restore NO bioavailability, increasing vasodilation and thus improving blood circulation in PVD patients. A randomized controlled trial [51] was conducted by N. Atalay Guzel et al. to investigate the effect of acute L-carnitine supplementation at two different doses on nitric oxide (NO) production and oxidative stress after exercise. Twenty-six healthy males (17-19 years of age) were given either 3 or 4 g L-carnitine in the form of a glass of fruit juice, and then all the participants ran on a treadmill. At the start of the test, the speed was set at 8 km/h, and after one hour, it was increased every three minutes by 1 km/h with one minute of rest before every heart rate increment to exhaustion. Venous blood samples were taken again five minutes after completion of the exercise test. The exact same test was repeated a week later on the same athletes; however, this time all subjects received the placebo, which was also a glass of fruit juice. Again, one hour after the same exercise protocol described above was performed, blood samples were collected immediately. For measurements of NOx (stable end products of NO), thiobarbituric acid (TBA) (a test measures malonaldehyde (MDA) produced due to the oxidation of fatty acids) and antioxidant glutathione (GSH) levels in plasma. The study findings indicate that the application of L-carnitine at a dose of 3 g acts as an efficient antioxidant, enhancing the levels of GSH and NOx and reducing the TBA level.

Moreover, L-carnitine has also been shown to have anti-inflammatory effects, as previously mentioned. PVD is closely related to inflammation, which plays a prominent role in atherosclerosis, the underlying cause of the disease, promoting endothelial injury and plaque formation.

A systemic review was conducted by Victor Kamoen et al. with 12 studies with a total number of 1423 randomized participants [52]. A majority of the included studies assessed PLC versus placebo (11 studies, 1395 participants), and one study assessed propionyl L-carnitine (PLC) versus L-carnitine (one study, 26 participants). We identified no RCTs that assessed PLC versus any other medication, exercise, endovascular intervention, or surgery. Participants received PLC 1 gram to 2 grams orally (nine studies) or intravenously (three studies) per day or placebo.

For the comparison of PLC versus placebo, there was a high level of both clinical and statistical heterogeneity due to study size, participants coming from different countries and centers, the combination of participants with and without diabetes, and the use of different treadmill protocols. We found a high proportion of drug company-backed studies. The overall certainty of the evidence was moderate.

For PLC compared with placebo, improvement in maximal walking performance (ACD) was greater for PLC than for placebo, with a mean difference in the absolute improvement of 50.86 meters (95% CI 50.34 to 51.38; nine studies, 1121 participants), or a 26% relative improvement (95% CI 23% to 28%). Improvement in pain-free walking distance (ICD) was also greater for PLC than for placebo, with a mean difference in absolute improvement of 32.98 meters (95% CI 32.60 to 33.37; nine studies, 1151 participants), or a 31% relative improvement (95% CI 28% to 34%). Improvement in ankle-brachial index (ABI) was greater for PLC than for placebo, with a mean difference in improvement of 0.09 (95% CI 0.08 to 0.09; four studies, 369 participants). Quality of life improvement was greater with PLC (MD 0.06, 95% CI 0.05 to 0.07; one study, 126 participants). Progression of disease and adverse events, including nausea, gastric intolerance, and flu-like symptoms, did not differ greatly between PLC and placebo.

For the comparison of PLC with L-carnitine, the certainty of evidence was low because this included a single, very small, cross-over study. Mean improvement in ACD was slightly greater for PLC compared to L‐carnitine, with a mean difference in absolute improvement of 20.00 meters (95% CI 0.47 to 39.53; one study, 14 participants) or a 16% relative improvement (95% CI 0.4% to 31.6%). We found no evidence of a clear difference in the ICD (absolute improvement 4.00 meters, 95% CI ‐9.86 to 17.86; one study, 14 participants); or a 3% relative improvement (95% CI ‐7.4% to 13.4%). None of the other outcomes of this review were reported in this study.

The combination of L-carnitine and the standard medication provides a new insight into this field for more improvement in symptoms and quality of life. A randomized, double-blind, placebo-controlled trial was conducted by Neil A. Goldenberg et al. to assess the effect of L-carnitine plus cilostazol versus cilostazol alone for the treatment of claudication in patients with peripheral artery disease with a sample size of 80 per arm [53]. In this trial, patients with stable IC were randomized to either L-carnitine 1 g or a matching placebo twice daily on a background of cilostazol. Treadmill and quality of life assessments were performed at baseline, 90, and 180 days. The key efficacy outcome was the natural-log-transformed (ln) ratio in peak walking time (PWT) at day 180 versus baseline. Treatment with cilostazol and L-carnitine was well tolerated in this study. In the modified intent-to-treat population (n = 145), the mean ln ratio in PWT was 0.241 for the cilostazol + L-carnitine treatment group versus 0.134 for the cilostazol + placebo treatment group (p = 0.076). This translates into mean increases of 1.99 and 1.36 min, respectively. In the per-protocol population (n = 120), the ln ratio in PWT was 0.267 for the cilostazol + L-carnitine treatment group versus 0.145 for the cilostazol + placebo treatment group (p = 0.048). There was an improvement in the quality of life (QOL) scores for the cilostazol + L-carnitine treatment group as well. Taken together, these data indicate a potential use for L-carnitine in combination with cilostazol for the treatment of IC. Larger trials are now needed to confirm the benefits that were observed here.

In conclusion, the results of the overall trials suggest the possible usefulness of L-carnitine in several cardiovascular pathological conditions, including cardiac failure, CAD, and PAD. Although these results should be confirmed by a large, randomized, experiment to validate a possible therapeutic role, considering the low cost and the good safety profile, L-carnitine therapy could be now deemed as a possible treatment in selected, high-risk patients.

Side effects of L-carnitine

Although the vast majority of users report benefits associated with few to no side effects, others have reported unpleasant gastrointestinal distress, a fishy body odor developed through its metabolism, cardiovascular issues, and even seizures.

The observed safe levels (OSL) indicate that the evidence of safety is strong at intakes up to 2000 mg/day of L-carnitine equivalents for chronic supplementation, and this level is identified as the OSL. Although much higher levels have been tested without adverse effects and may be safe, the data for intakes above 2000 mg/day are not sufficient for a confident conclusion of long-term safety [54].

The majority of users who report gastrointestinal side effects reported that they are dose-dependent [7]. The fishy body odor is thought to be caused by the body’s metabolism of L-carnitine into trimethylamine (TMA) and is thought to be dose-dependent as well [55]. Fish odor syndrome is a syndrome in which a lack of, or diminished, flavin-dependent monooxygenase (FMO3) activity (which converts TMA to TMAO) leads to the accumulation of TMA in the blood [55].

More importantly, human clinical observations and preclinical animal studies revealed that TMAO is a key mediator of atherogenesis. Five possible pathways through which TMAO may modulate pro-atherosclerotic mechanisms have been proposed [55]. A meta-analysis study was conducted by Luigina Guasti et al. [56] evaluating the association between TMAO and CVD events. A total of three studies (923 high CVD risk) were pooled together. A high TMAO level is related to both major adverse cardiovascular risk (RR = 2.05; 95% CI 1.61-2.61) and all-cause mortality (RR = 3.42; 95% CI 2.27-5.15).

Further systemic review [57] was conducted by Gabriele Schiattarella et al., assessing TMAO as a new potentially important cause of increased cardiovascular risk. This meta-analysis included 17 clinical studies in total (enrolling 26,167 subjects) and had a median of 4.3 ± 1.5 years of mean follow-up. High plasma TMAO concentrations were associated with increased incidence of all-cause mortality (14 studies for 16 cohorts enrolling 15,662 subjects, hazard ratio: 1.91; 95% confidence interval: 1.40-2.61, P < 0.0001, I2 = 94%) and major adverse cardio and cerebrovascular events (MACCE) (five studies for six cohorts enrolling 13,944 subjects, hazard ratio: 1.67, 95% confidence interval: 1.33-2.11, P < 0.00001, I2 = 46%). In the dose-dependence meta-analysis, for a 10 μmol/L increment of TMAO concentration, the relative risk for all-cause mortality increased by 7.6% (summary relative risk: 1.07, 95% CI (1.04-1.11), P < 0.0001; based on seven studies). This was the first systematic review and meta-analysis demonstrating the positive dose-dependent association between TMAO plasma levels and increased cardiovascular risk and mortality.

Furthermore, L-carnitine is reported to lower the threshold for seizures in some individuals, especially those with a history of seizures, but no evidence to support this alleged effect has been found [58].

Discussion around the existing research on L-carnitine in cardiovascular health

In conclusion, when all the existing data is analyzed, L-carnitine supplementation and cardiovascular health appear to be quite a complicated issue, rather than a matter of yes or no, black or white, good or bad. There is a marked tendency for L-carnitine supplementation to reduce both mortality and arrhythmias post-AMI, albeit (as with other data) this is not universally true, and many studies had a lot of limitations as previously discussed. The blend of good and bad effects of L-carnitine supplementation, together with the methodological differences in the findings between studies, suggest that furthermore, rigorous research is necessary to clarify exactly what role, if any, L-carnitine supplementation should have in the prevention and treatment of CVD.

Possibly the most impressive data relate to L-carnitine as a therapy for improving outcomes in acute myocardial infarction. Many trials and meta-analyses have purported to show that L-carnitine supplementation can reduce mortality and arrhythmia events.

Despite these findings, the evidence is by no means uniformly positive. For instance, some studies have failed to show any benefit of L-carnitine supplementation on outcomes such as the incidence of heart failure or myocardial reinfarction (CEDIM-2 trial). Thus, while one systematic review noted impressive reductions in mortality and arrhythmia, it did not find corresponding reductions in heart failure or recurrent myocardial events [47]. Such variability helps to explain the complexity of L-carnitine’s mechanisms of action and its inconsistent effects across cardiovascular endpoints.

The proposed potential mechanisms for L-carnitine’s cardiovascular benefits have been the focus of much research. L-carnitine increases mitochondrial energy metabolism and plays a specific role in minimizing myocardial ischemia and replacing the depleted carnitine, which can occur during the metabolic stress of an acute myocardial infarction. It also appears to help minimize left ventricular remodeling and thus the size of infarcts, improving cardiac function. How L-carnitine fulfills these potential functions is not completely understood, and a better comprehension can be realized through further mechanistic studies.

Secondly, the intestinal metabolism of L-carnitine is thought to generate trimethylamine-N-oxide (TMAO), a compound that has been linked with faster atherosclerosis progression. Taken together, this dichotomy suggests the possibility that, in patients recovering from an acute myocardial infarction (AMI), L-carnitine may have ‘protective’ effects in the short-term but long-term risk-enhancing effects on atherosclerosis, particularly if administered via high-dose supplements. Given the pro-atherogenic pathway, the net clinical benefit of L-carnitine must be carefully weighed against its cardioprotective effects.

The picture is further muddied by limitations in the studies, and different study designs, e.g., variations in patient populations, dosages, treatment durations, and outcomes, which make it impossible to synthesize a final answer to whether L-carnitine works.

The issue continues to captivate science, as evidenced by ongoing trials such as "Effect of L-carnitine on Biomarkers of Myocardial Reperfusion Injury in Patients With STEMI," expected to conclude by the end of next year. The study aims to evaluate the efficacy and tolerability of L-carnitine on biomarkers of myocardial reperfusion injury in patients with ST-segment elevation myocardial infarction (STEMI) undergoing primary PCI by the assessment of nitrotyrosine as an oxidative stress marker and matrix metallopeptidase-2 (MMP-2) as a cardiac fibrosis marker (ClinicalTrials.gov ID NCT06564909, Sponsor Ain Shams University [59].

Future research directions for the role of L-carnitine in cardiovascular health

This body of preclinical and clinical evidence allows us to craft our next step. The gaps in our knowledge and future questions represent a roadmap to the next level of research that can further elucidate L-carnitine’s impact on cardiovascular health.

Knowledge gaps in the literature help point the way to future research. While a large number of randomized trials have looked into the use of L-carnitine to prevent mortality and ventricular arrhythmias after AMI, the question of whether L-carnitine can prevent heart failure and myocardial re-infarction is still somewhat nebulous. This suggests that trials designed specifically to look into those endpoints are warranted. At a cellular and molecular level, more work is needed to explore the mechanisms of how L-carnitine could exert its action, whether due to its impact on mitochondrial energy metabolism, its potentially anti-ischemic qualities, or its effect on left ventricular remodeling.

Moreover, given that its toxic metabolite, trimethylamine-N-oxide (TMAO), facilitates low-density lipoprotein (LDL) uptake into the arterial wall, promoting atherosclerosis, there is presently little evidence to support the long-term safety of L-carnitine supplementation. Looking forward to future research, it needs to be determined whether the efficacy of L-carnitine outweighs the potential cardiovascular hazards in appropriate patient populations that will maximize its cardiovascular benefits without incurring significant harm.

Suggested areas for future study were detailed groups, which include extensive clinical trials evaluating wider cardiovascular disorders and population groups, to work out the appropriate dose and maximum course of treatment to maximize therapeutic effects and minimize unwanted effects and to integrate genetic, lifestyle, and disease severity factors into the study design to understand if these factors might affect treatment. This would help to understand if there are specific patient populations that will benefit from L-carnitine supplementation. It’s important to understand that this medication is not only effective when taken as a supplement by those with heart disease, but it also will help cardiologists determine more individualized treatment strategies for patients who might benefit from it.

Second, mechanistic studies are necessary to understand how L-carnitine works under the cellular microscope. Studies could delve more deeply into the compound’s effects on mitochondrial dynamics, energy metabolism, and oxidative stress, which appear to contribute to its benefit in cardiovascular disease. Investigations such as these could reveal new therapeutic targets for improving cardiac metabolic pathways and devising new interventions for cardiovascular management. A better understanding of how L-carnitine interacts with other metabolic substrates and the existing pharmacological therapies currently available for use in patients could lead to optimal strategies for integrating L-carnitine with existing therapy.

In conclusion, although it is clear that cardiovascular benefits can be achieved with L-carnitine supplementation, more research needs to be done to address many gaps. Future research should determine the long-term outcomes and safety profile. In addition, it is still not clear how the benefits of L-carnitine are mediated or what is actually responsible for the benefits. Future well-designed clinical trials will better answer these questions, providing further guidance for a therapeutic utility for L-carnitine and potentially improving patient care.

## Conclusions

In conclusion, the actions of L-carnitine on the cardiovascular system are complex and explain the potential advantages and disadvantages of cardiovascular action of this drug. The available clinical literature represents promising data that shows that the administration of L-carnitine may dramatically decrease mortality, ventricular arrhythmia, and anginal symptoms in high-risk patients, especially after an acute ischemic event such as an acute myocardial infarction. Despite these encouraging findings, the evidence is fraught with inconsistencies and methodological limitations.

Finally, there’s a need for larger-scale, well-designed, randomized controlled trials to answer these shortcomings and inconsistencies of existing studies. Future clinical trials should be performed in much larger patient cohorts with attention to the longer-term outcomes and safety profile of L-carnitine supplementation over prolonged periods. The optimal dosing regimens and duration of supplementation are also important aspects to be further explored regarding the risk versus curative benefits. By addressing these current knowledge gaps, the research community will be able to provide clinicians and their patients with more definitive data on the evidence-based integration of the paradigms of cardiovascular disease prevention and treatment.
